# Oral frailty and its influencing factors among ICU patients with oral endotracheal intubation: a cross-sectional study

**DOI:** 10.3389/fmed.2025.1650313

**Published:** 2025-10-02

**Authors:** Min Shi, Lei Li, Huaixiang Zhai

**Affiliations:** ^1^Critical Care Medicine, The First People’s Hospital of Lianyungang, Lianyungang, China; ^2^Lianyungang Disease Prevention Control Center, Lianyungang, China

**Keywords:** ICU, oral endotracheal intubation, oral frailty, influencing factor, cross-sectional study

## Abstract

**Objective:**

To investigate the level and factors associated with oral frailty among ICU patients with oral endotracheal intubation, and to provide references for the construction of targeted nursing intervention programs in the future.

**Methods:**

In this single-centre, cross-sectional study, a total of 226 patients with oral endotracheal intubation in ICU were selected by convenience sampling method. General data questionnaire, the Oral Frailty Index-8, the Oral Health Assessment Tool, the Nutritional Risk Screening 2002 and the Frail Scale were used for the study.

**Results:**

The scores of oral frailty in ICU patients with oral endotracheal intubation were 3.00 (2.00, 4.00), of which 36.73% (83/226) were high risk. Age, marital status, duration of oral endotracheal intubation and oral health level were the influencing factors of oral frailty in ICU patients with oral endotracheal intubation (all *p* < 0.05).

**Conclusion:**

The risk of oral frailty among ICU patients with oral endotracheal intubation is prevalent. In addition to airway management, medical staff should pay attention to the level of oral frailty according to the patient’s specific situation, and do a good job of prevention and control management to reduce the risk of oral frailty.

## 1 Introduction

Oral endotracheal intubation (1) is an important means of life support in the ICU. It can maintain the patency of the patient’s respiratory tract and improve oxygenation status. However, at the same time, it affects the oral environment. In severe cases, it may damage oral functions and increase the physical and mental burden of patients (2). Study (3) shows that poor oral health is one of the common problems among patients with invasive mechanical ventilation in the ICU. The establishment of artificial tracheas interferes with the normal oral functions of patients. Patients have limited chewing function, reduced oral saliva, decreased self-cleaning ability, accumulation of airway secretions, and even cause swallowing dysfunction (4). Relevant studies (5) showed that patients with tracheal intubation are prone to complications such as respiratory tract infections due to misaspiration. Studies (6, 7) have reported that patients with long-term tracheal intubation connected to a ventilator for assisted breathing showed functional impairment such as choking when drinking water or drooling 4 h after extubation. Poor oral health of patients is prone to increase their risk of frailty (8). It may even cause physical, psychological and social impairments, thereby leading to a decline in the quality of life related to oral health in patients (9). Therefore, the current situation of the quality of life related to oral health in patients should be given more attention (10). Effective oral care management is regarded as one of the important measures to prevent related complications in patients with mechanical ventilation (11). Relevant studies (12) show that the oral health care practice programs in our country are still in the development stage.

Studies show that the incidence of oral frailty in China is higher than that in developed countries such as Japan (13). This might be due to differences in healthcare, culture, etc., or perhaps because of variations in research methodologies (14). Oral frailty has a significant impact on the health, possibly increasing the risk of physical frailty, disability and death, and is closely related to muscle loss, cognitive decline and deterioration of quality of life (15–17). By assessing the oral frailty of patients, it can help us identify the risk of their death at an early stage (18). Studying the occurrence of oral frailty and intervention measures is of great significance for improving the oral health of patients, enhancing their quality of life and delaying aging. At present, China pays more attention to the physical, psychological and cognitive frailty of patients, but the research on oral frailty is relatively insufficient. Dibello et al. (19) pointed out that decreased chewing ability, difficulty in swallowing and tooth loss are the main characteristics of oral frailty. These problems can significantly affect the daily living ability of patients and increase the risk of physical frailty. Another study also found that poor oral health is significantly associated with frailty, with tooth loss and chewing difficulties being key risk factors (20). Research (21) shows that the oral environment and function affect the recovery of the body and are even related to the daily activities of patients after discharge. Therefore, paying attention to oral conditions can not only effectively prevent complications such as ventilator-associated pneumonia (22), but also be conducive to promoting the recovery of patients.

Foreign scholars have paid attention to oral frailty earlier and have carried out related research, while the attention to oral frailty in China is also gradually increasing (23). Currently, the main focus is on the incidence and influencing factors of oral frailty, etc. Studies (24–28) show that the incidence of oral frailty in China ranges from 25.19 to 64.3%. Research (29) shows that oral frailty is age-related. With the increase of age, the occlusal function, chewing function and swallowing function of the elderly all decline to varying degrees. Meanwhile, living habits can also affect the degree of oral frailty. People who live alone or often eat alone have a higher risk of developing oral frailty (30). Different research subjects have different levels of oral frailty, and the focus of attention also varies. For instance, among elderly patients with long-term T2DM duration of more than 10 years, particular attention should be paid to fewer functional natural teeth, suboptimal dental restoration status, elevated FBG levels, and coexisting cognitive impairment (31). There are numerous risk factors for oral frailty in patients. It is very necessary to discover and warn of the risk factors of oral frailty in patients in a timely manner. Relevant research suggests incorporating oral health into physical health assessment, which is of great significance for preventing the overall progression of individual frailty (32). Therefore, formulating effective intervention measures for oral frailty in the future to prevent oral frailty in patients is of great significance for healthy.

Therefore, from the perspective of patients with oral endotracheal intubation in the ICU, this study explores the level of oral frailty and its influencing factors, with the aim of enhancing the attention of medical staff to the oral health level of patients with oral endotracheal intubation in the ICU in clinical work. At the same time, it also provides a reference for formulating intervention measures to improve oral frailty in the future.

## 2 Materials and methods

### 2.1 Study design and participants

Convenience sampling method was used to select patients with oral endotracheal intubation in the ICU of a tertiary general hospital in Lianyungang City, Jiangsu Province, China, from August 2024 to January 2025 as the study subjects. All patients provided a signed written informed consent form for the use of their data. The study complied with the principles outlined in the Declaration of Helsinki and was approved by the Ethics Committee of the Hospital (KY-20250514001-01).

Inclusion criteria included: (1) age ≥18 years old; (2) When awake /RASS score was 0, the duration of oral endotracheal intubation was ≥48 h; and (3) The patient’s condition is stable and they can communicate through verbal or non-verbal means (such as writing), and independently complete the result responses throughout the process. Exclusion criteria included: (1) presence of cognitive impairments or mental disorders; (2) comorbidity with acute or severe diseases.

According to the requirements of statistical variable analysis, the required sample size should be 5–10 times the number of variables (33), calculation formula: Number of variables × (5 to 10) + Number of variables × (5 to 10) × inefficiency. In this study, there are 19 variable factors, and Considering the inefficiency of 10% questionnaire, the required sample size is at least 105 cases, and a total of 226 patients were investigated in the end.

### 2.2 Research tools

#### 2.2.1 General information questionnaire

The researcher developed a general information questionnaire to investigate the general sociological information and related situations of the research participants through literature reading and data searching. The content included gender, age, marital status, smoking history, drinking history, education level, average monthly income, occupation, Body Mass Index (BMI), number of chronic co-morbidities, length of stay in ICU, duration of oral endotracheal intubation, whether surgical treatment was performed, whether sedative medication was used, whether dry mouth was present, and whether denture was present.

#### 2.2.2 Oral Frailty Index-8

This scale was developed by Tanaka et al. (34). There are a total of 8 items, mainly covering five aspects: chewing ability, swallowing function, presence of dentures, oral health care behaviors, and social activities, which were scored by “yes” and “no.” Entries 1–3 “yes” were scored as 2 points and “no” as 0 points, entries 4 ~ 5 “Yes” scored 1 point, “No” scored 0 points, and entries 6–8 “Yes” was scored as 0 points and “No” was scored as 1 point, and the total score ranges from 0 to 11 points. The higher the score, the worse the condition of oral debility (35), where 0–2 is low risk, 3 is medium risk, and ≥4 is high risk (24). The Cronbach’s α coefficient for the scale in this study was 0.699. The items of this scale are concise, the data is easy to collect, and it has good predictability (36). Even without the direct participation of oral professionals, medical staff can accurately assess it (37).

#### 2.2.3 Oral Health Assessment Tool

The Oral Health Assessment Tool (OHAT) was Chineseized by Wang Jieqiong (38) and others on the basis of the revised scale by Chalmers (39) and others, and includes 8 entries for lips, tongue, gingival tissues, saliva, natural teeth, denture, oral cleanliness, and dental pain. Adopt Likert 3-level scoring method, from “healthy” to “unhealthy” score 0 ~ 2., with a total score of 0 to 16, with higher scores indicating poorer oral health. The Cronbach’s α coefficient for this scale was 0.871 in this study.

#### 2.2.4 Nutritional Risk Screening 2002

This scale was developed by Kondrup et al. (40). It was used to assess the risk of malnutrition in the study population, including three aspects: disease severity (0–3 points), nutritional impairment (BMI, recent weight changes and changes in eating, 0–3 points), age (≥70 years: “yes” scores 1, “no” scores 0), and a total score of 0–7 points, with a score of ≥3 indicating the presence of malnutrition (41).

#### 2.2.5 Frail Scale

The scale is localized by Wei Yin (42) and others based on the frail screening tool proposed by the a European, Canadian and American Geriatric Advisory Panel (43), including five aspects, such as fatigue, resistance movement, walking difficulty, multi-disease coexistence and weight change, each item “yes” counts 1 point, “no” counts 0 points, and the total number is 0 to 5 points. 0 is classified as low risk, 1–2 as medium risk, and ≥3 as high risk. The Cronbach’s *α* coefficient of the scale in this study was 0.891.

### 2.3 Data collection

The on-site survey was conducted by uniformly trained researchers, and one-on-one investigation is conducted with unified and colloquial instructions to explain the purpose and significance of this survey. The questionnaires were filled out on the spot and collected on the spot, and the completeness of the data was checked in time to ensure the authenticity and validity of the questionnaires. This study investigated the oral frailty level of patients 0 to 12 h after tracheal intubation removal, lasting for 10 to 30 min. Selecting this time point for the measurement of outcome indicators can not only avoid the impact of intubation on the patient’s condition and expression, but also maintain the timeliness of the effect of oral tracheal intubation on the patient’s oral frailty. Throughout the process, the patients were covered with bed curtains to protect their privacy. For research subjects with lower educational attainment or Poor audio-visual quality, assistance will be provided in the form of questions and answers.

### 2.4 Statistical analysis

The statistical analysis of this study was conducted using SPSS 26.0. The quantitative data were analyzed using frequencies and percentages. The measurement data conforming to normal distribution were expressed as mean ± standard deviation, and the two independent samples *t*-test was used for inter-group comparison. Those that did not conform to normal distribution were represented by median and quartile. Mann–Whitney U test was used for comparison between the two groups of data, Kruskal-Wallis H test was used for comparison between multiple groups of data, and Spearman correlation analysis was used for correlation. The variables with statistical significance were taken as independent variables by single factor analysis, and multivariate linear regression analysis was used for multivariate analysis. *p* < 0.05 was considered statistically significant.

## 3 Results

### 3.1 Characteristics of participants

A total of 226 patients were recruited for this study, including 108 males (47.8%) and 118 females (52.2%). Additional demographic details are shown in Table 1.

**Table 1:** Comparison of oral frailty levels in ICU patients with oral tracheal intubation of different characteristics.

Characteristics	*N* (Percentage/%)	Z/H	*P*
Gender		−1.166	0.244
Male	108 (47.8%)		
Female	118 (52.2%)		
Marital status		9.511	0.009
Married	207 (91.6%)		
Unmarried	2 (0.9%)		
Divorced/widowed	17 (7.5%)		
Smoking history		−1.863	0.062
Yes	63 (27.9%)		
No	163 (72.1%)		
Drinking history		−0.342	0.732
Yes	82 (36.3%)		
No	144 (63.7%)		
Education level		10.071	0.018
Primary school and below	57 (25.2%)		
Junior high school	84 (37.2%)		
High school/technical secondary school	66 (29.2%)		
College/university degree or above	19 (8.4%)		
Average monthly income (yuan)		3.251	0.197
<1,000	59 (26.1%)		
1,000 ~ 5,000	79 (35.0%)		
>5,000	88 (38.9%)		
Occupation		49.927	<0.001
Employed	43 (19.0%)		
Retirement	151 (66.8%)		
Unemployed/Freelancer/self-employed	32 (14.2%)		
Body Mass Index (kg/m^2^)		0.065	0.968
<18.5	61 (27.0%)		
18.5 ~ 23.9	120 (53.1%)		
≥24	45 (19.9%)		
Number of chronic co-morbidities		5.281	0.071
0	25 (11.1%)		
1	81 (35.8%)		
≥2	120 (53.1%)		
Whether surgical treatment was performed		−2.556	0.011
Yes	59 (26.1%)		
No	167 (73.9%)		
Whether sedative medication was used		−1.825	0.068
Yes	119 (52.7%)		
No	107 (47.3%)		
Whether dry mouth was present		−0.858	0.391
Yes	120 (53.1%)		
No	106 (46.9%)		
Whether denture was present		−0.819	0.413
Yes	90 (39.8%)		
No	136 (60.2%)		
Nutritional risk		−3.827	<0.001
Yes	86 (38.0%)		
No	140 (62.0%)		
Frailty risk		29.234	<0.001
Low	8 (3.5%)		
Medium	107 (47.4%)		
High	111 (49.1%)		

### 3.2 Univariate analysis of ICU patients with oral endotracheal intubation with different characteristics

As shown in Table 1, items with statistically significant differences in marital status, education level, occupation, whether surgical treatment was performed, nutritional risk, frailty risk.

### 3.3 Oral frailty score of ICU patients with oral endotracheal intubation, and correlation analysis with age, length of stay in ICU, duration of oral endotracheal intubation and oral health level

The results of the analysis showed that the oral frailty score of ICU patients with oral endotracheal intubation was 3.00 (2.00, 4.00) points, with 28.32% (64 patients) at low risk, 34.96% (79 patients) at medium risk, and 36.72% (83 patients) at high risk. Spearman correlation analysis showed that the correlation coefficients of oral frailty score with age, length of stay in ICU, duration of oral endotracheal intubation and oral health level were *r* = 0.654, 0.515, 0.560 and 0.505, respectively, all *p* < 0.001. Detailed results are provided in Table 2.

**Table 2:** The correlation between oral frailty score and age, length of stay in ICU, duration of oral tracheal intubation, and oral health level score (*r* value).

Item	Age	Length of stay in ICU	Duration of oral tracheal intubation	Oral health level
Oral frailty	0.654	0.515	0.560	0.505

All *P* < 0.001.

### 3.4 Influencing factors of oral frailty among ICU patients with oral endotracheal intubation

The oral frailty score was used as the dependent variable and the variables with statistically significant differences in the univariate analysis were used as independent variables for multiple linear regression analysis. Age, length of stay in the ICU, duration of oral tracheal intubation and oral health level were brought in as original values. The assignment of values for marital status, educational level, occupation, whether surgical treatment was performed, nutritional risk and frailty risk is as follows (marital status:1 = Married, 2 = Unmarried, 3 = Divorced/widowed; educational level:1 = Primary school and below, 2 = Junior high school, 3 = High school/technical secondary school, 4 = College/university degree or above; occupation:1 = Employed, 2 = Retirement, 3 = Unemployed/Freelancer/self-employed; whether surgical treatment was performed:1 = Yes, 2 = No; nutritional risk:1 = Yes, 2 = No; frailty risk:1 = Low, 2 = Medium, 3 = High). Multiple linear regression showed that age, marital status, duration of tracheal intubation and oral health were the influencing factors of oral frailty in ICU patients with oral endotracheal intubation. Table 3 presents the multiple linear regression analysis results.

**Table 3:** Multivariate analysis of oral frailty in patients with oral tracheal intubation in the ICU.

Independent variable	*β*	SE	*β*′	*t*	95%CI	*P*
Constant	−2.93	0.764	–	−3.837	(−4.436 ~ −1.425)	<0.001
Age	0.068	0.011	0.444	6.133	(0.046 ~ 0.090)	<0.001
Marital status	0.458	0.172	0.113	2.663	(0.119 ~ 0.797)	0.008
Education level	0.037	0.103	0.016	0.354	(−0.167 ~ 0.240)	0.723
Occupation	−0.081	0.162	−0.022	−0.497	(−0.401 ~ 0.239)	0.620
Length of stay in ICU	−0.021	0.025	−0.104	−0.844	(−0.071 ~ 0.028)	0.400
Duration of oral endotracheal intubation	0.142	0.029	0.595	4.842	(0.084 ~ 0.200)	<0.001
Whether surgical treatment was performed	0.143	0.208	0.029	0.685	(−0.268 ~ 0.553)	0.494
Oral health	0.067	0.029	0.145	2.286	(0.009 ~ 0.125)	0.023
Nutritional risk	−0.097	0.197	−0.022	−0.494	(−0.485 ~ 0.291)	0.622
Frailty risk	−0.102	0.196	−0.027	−0.519	(−0.487 ~ 0.284)	0.604

## 4 Discussion

The present study utilized a cross-sectional design to investigate the degree of oral frailty and its influencing factors among ICU patients with oral endotracheal intubation. The study’s findings revealed that ICU patients with oral endotracheal intubation exhibited oral frailty scores of 3.00 (2.00, 4.00) points, with 28.32% (64 patients) at low risk, 34.96% (79 patients) at medium risk, and 36.72% (83 patients) at high risk. The proportion of high-risk patients was lower than that of the survey results of the elderly in the community by Wang Lin et al. (26) (59.2%), which may be due to differences in research objects; ICU nurses use oral care solution every day to provide oral care for patients by wiping, rinsing and other reasonable ways to help patients clean their mouths and improve their comfort; Healthcare professionals provide patients with targeted treatment and care measures and 24 h care to facilitate their early recovery stage. However, the results of this study are higher than those of Irie et al. (44) (28.1%), which may be attributed to regional differences in the study subjects and differences in the study base. The study (45) shows that the elderly in Japan have a high awareness of oral health and visit dental clinics regularly. However, patients with oral catheterization in ICU are affected by many factors. Endotracheal catheterization and dental pads in the mouth lead to limited chewing function, stranded swallowing function, inability to eat orally, weakened oral self-purification ability, which is not conducive to the operation of oral care, and easy to induce oral infection and even ventilator-associated pneumonia (46). In addition, due to the critical condition of patients with oral endotracheal intubation in ICU, patients and their families pay more attention to the patient’s condition and treatment effect, and then ignore oral problems. This suggests that healthcare professionals should promptly identify the risk of oral frailty among ICU patients with oral endotracheal intubation, carry out oral frailty risk screening when the patient’s condition permits, and provide relevant professional knowledge and nursing care measures for patients in a targeted manner. For patients who have already experienced oral frailty, effective interventions, such as attempting to integrate appropriate nursing techniques of Chinese medicine, can help patients slow down the decline of oral function, reduce the risk of oral frailty among ICU patients with oral endotracheal intubation, improve the oral status and the quality of life, and promote the recovery of the patients.

The results of this study show that age is an influencing factor for oral frailty in ICU patients with oral endotracheal intubation, and the older the patients are, the higher the level of oral frailty is, which is consistent with the results of Tu Hangjia et al. (25). This may be due to the deterioration of body function with the increasing age of patients, and the worse oral environment due to oral catheterization, which leads to the decline of oral function. Oral weakness occurs, and decreased oral function is prone to malnutrition and muscle loss (47). Studies (48) have shown that oral function exercise as well as increased protein intake are beneficial in improving oral function. Therefore, medical staff should pay attention to the oral function of patients with oral tube intubation, especially older patients, and should follow the doctor’s advice to remove the tracheal intubation as soon as possible if the condition permits, which can not only reduce the risk of infection, but also help improve the oral status of patients. The patient was instructed to eat reasonably after extubation and gradually increase protein intake to improve the patient’s nutritional status and then reduce the risk of oral weakness.

The analysis results found that marital status was significantly associated with a higher risk of oral frailty in ICU patients with oral endotracheal intubation. Compared with patients with spouses, patients without spouses had a higher risk of oral frailty, which was similar to the results of previous studies (30), and single eaters were more likely to suffer from oral frailty (49). Nagayoshi et al. (50) showed that marital status correlates with oral debility, in which men with a partner have better oral function, probably because the companionship of their spouses enriches their daily life and improves their tongue and lip movement during communication (51). Related studies (52) have shown that oral debility is detrimental to an individual’s physical and mental health as well as social development, and the patients in this study received love and support from their partners or spouses during regular visits, which was important for their physical and mental recovery. While our data suggest an association, the underlying mechanisms remain unexplored. Future studies incorporating measures of social support, psychological stress, and health behaviors are warranted to elucidate the potential pathways linking marital status and oral health outcomes. In the future, it is recommended that medical staff guide patients to carry out oral function rehabilitation exercises in a planned way (53), and encourage family members to participate in them, which is conducive to reducing the risk of oral weakness.

The results of this study show that the duration of oral endotracheal intubation is an influencing factor for oral frailty in ICU patients with oral tracheal intubation, which is similar to the results of previous studies (12). The duration of oral tracheal intubation is correlated with oral frailty. However, as this study was a cross-sectional design, this result only indicates an association between the two. It cannot be inferred that the longer the time of oral tracheal intubation, the more likely oral frailty will occur, nor can the influence of reverse causality or potential confounding factors be ruled out. The duration of oral tracheal intubation is associated with the risk of complications, such as pressure injury, ventilator-associated pneumonia, etc. (54). In addition, patients with critical illness and decreased oral immunity are prone to various oral diseases, aggravating oral weakness and not conducive to recovery of the disease. Studies (55) have shown that oral health affects body function, and poor oral health may lead to malnutrition, muscle atrophy, etc., and may even trigger death (56). As ICU medical staff focus on patient recovery and treatment and nursing effects, and pay little attention to oral health care (12), it is suggested to strengthen the training of oral expertise and skills in the future, and encourage dental specialists to join the treatment and nursing of ICU patients with severe illness, so as to develop a multidisciplinary collaborative medical work mode. Especially for patients with oral tube intubation in ICU, medical staff should assess the patient’s condition in real time, conduct off-line training if the condition permits, and pull out the tracheal intubation as soon as possible (57), so as to avoid oral weakness to the greatest extent.

In this study, it was found that oral health level was an influential factor for oral frailty in ICU patients with oral endotracheal intubation, and the poorer the oral health status, the higher the oral frailty level, which was consistent with the results of Li Yi et al. (27). Oral health conditions mainly include lips, tongue, gum tissue, saliva, natural teeth, dentures, oral cleanliness, and toothache. Oral catheterization may affect the patient’s oral environment, such as compression of oral tissue and interference with oral saliva secretion, and tooth loss may also occur, leading to decreased patient comfort and limited oral function (58). At the same time, patients cannot clean their mouths by themselves during the process of oral tube intubation, and mainly rely on oral care provided by medical staff. Once oral cleaning is incomplete, it is easy to cause patients’ oral flora imbalance, which will lead to oral problems and accelerate oral weakness. Studies (59) have shown that effective oral management can improve the oral environment of patients with oral tube intubation, while different oral care methods have different effects on the oral environment of patients with oral tube intubation in ICU (60, 61). Therefore, healthcare professionals should take a reasonable approach to assess the oral condition of patients with tracheal intubation (2), construct a personalized oral care plan for them (62), provide perfect and comprehensive nursing measures, improve the oral environment, and enhance the quality of life. In addition, based on the actual situation of oral care for patients with oral catheterization in ICU in China and combined with oral nursing practice experience at home and abroad, oral nurses in intensive care can be developed to provide professional and perfect oral health care services for patients, thus reducing the occurrence of oral weakness in patients with oral catheterization.

There are several limitations to this cross-sectional study. First, we adopted a convenient sampling method, which might affect the external validity of the research results and introduce selection bias. Specifically, our sample size is relatively small, and all the samples are from a tertiary hospital, which may not fully represent the broader group of ICU patients with oral tracheal intubation. Moreover, patients with different severity of the disease have different perceptions of oral frailty, which may lead to differences between the sample and the overall population. Second, the study has several limitations inherent to cross-sectional research, such as subjective bias, which may not fully reflect the actual situation. There may be recall bias in patients’ self-reports, thus leading to overestimation. Although this assessment tool mainly relies on self-reported outcomes by patients, the scale includes elements of oral professional examination, which can accurately assess even without the direct participation of oral professionals, such as “Are there dentures/prosthetics?” Third, the scope of this study was limited to a certain moment, and no longitudinal tracking of outcome indicators was conducted. It failed to dynamically reflect the changes in oral vulnerability of ICU patients who received oral tracheal intubation, nor could it identify subgroups with different needs. Finally, no other factors that might affect ICU patients with oral tracheal intubation, such as baseline comorbidities and duration of sedation, were examined. Although we adjusted for multiple potential confounding factors, due to data availability issues, we were unable to adjust for baseline comorbidities and sedation duration, which may affect the accuracy of the results. Future research should prioritize the collection of these key clinical data for more thorough analysis. Despite these limitations, this study revealed the oral frailty among ICU patients with oral endotracheal intubation and its influencing factors, which has several implications for future research and practice. In the future, the research team will strive to overcome limitations, adopt more rigorous sampling methods, add objective outcome indicators, and collect longitudinal data, etc., to verify our findings. And this study illustrates the need for interventions to improve the oral frailty among ICU patients with oral endotracheal intubation. Future research should further expand the sample size and conduct multi-center investigations. At the same time, a more in-depth factor analysis of this scale should be carried out, and methods such as test–retest reliability should be considered to comprehensively evaluate its reliability. Therefore, in the future, the research team hopes to be able to find more effective and economical intervention strategies based on the existing research foundation and around the research hotspots of this topic (63–66).

## 5 Conclusion

The results of this study showed that the risk of oral frailty was common in patients with oral endotracheal intubation in ICU. Older age, no spouse, longer intubation time, and poor oral health are associated with an increased incidence of oral frailty in patients with oral tracheal intubation in the ICU. However, before clarifying the causal relationship, clinical decisions still need to be made with caution. It is suggested that ICU medical staff should pay attention to the assessment of oral frailty risk in patients with oral catheterization, pay special attention to key groups, formulate personalized intervention plans for patients with different characteristics, improve the oral health of patients with oral catheterization in ICU, reduce the risk of oral frailty, and promote early recovery. This study was a cross-sectional study and could not dynamically assess the changes in patients with oral frailty. In the future, multi-center, large-sample longitudinal studies can be carried out to further explore the level of oral frailty in critically ill patients.

## Data Availability

The raw data supporting the conclusions of this article will be made available by the authors, without undue reservation.
